# Water Quality Is a Poor Predictor of Recreational Hotspots in England

**DOI:** 10.1371/journal.pone.0166950

**Published:** 2016-11-22

**Authors:** Guy Ziv, Karen Mullin, Blandine Boeuf, William Fincham, Nigel Taylor, Giovanna Villalobos-Jiménez, Laura von Vittorelli, Christine Wolf, Oliver Fritsch, Michael Strauch, Ralf Seppelt, Martin Volk, Michael Beckmann

**Affiliations:** 1 University of Leeds, School of Geography, Leeds, LS2 9JT, United Kingdom; 2 University of Leeds, School of Earth and Environment, Leeds, LS2 9JT, United Kingdom; 3 University of Leeds, School of Biology, Leeds, LS2 9JT, United Kingdom; 4 UFZ, Helmholtz Centre for Environmental Research, Department of Environmental and Planning Law, Permoserstraße 15, 04318 Leipzig, Germany; 5 UFZ, Helmholtz Centre for Environmental Research, Department of Economics, Permoserstraße 15, 04318 Leipzig, Germany; 6 UFZ, Helmholtz Centre for Environmental Research, Department of Computational Landscape Ecology, Permoserstraße 15, 04318 Leipzig, Germany; 7 Institute of Geoscience & Geography, Martin-Luther-University Halle-Wittenberg, 06099 Halle (Saale), Germany; Peking University, CHINA

## Abstract

Maintaining and improving water quality is key to the protection and restoration of aquatic ecosystems, which provide important benefits to society. In Europe, the Water Framework Directive (WFD) defines water quality based on a set of biological, hydro-morphological and chemical targets, and aims to reach good quality conditions in all river bodies by the year 2027. While recently it has been argued that achieving these goals will deliver and enhance ecosystem services, in particular recreational services, there is little empirical evidence demonstrating so. Here we test the hypothesis that good water quality is associated with increased utilization of recreational services, combining four surveys covering walking, boating, fishing and swimming visits, together with water quality data for all water bodies in eight River Basin Districts (RBDs) in England. We compared the percentage of visits in areas of good water quality to a set of null models accounting for population density, income, age distribution, travel distance, public access, and substitutability. We expect such association to be positive, at least for fishing (which relies on fish stocks) and swimming (with direct contact to water). We also test if these services have stronger association with water quality relative to boating and walking alongside rivers, canals or lakeshores. In only two of eight RBDs (Northumbria and Anglian) were both criteria met (positive association, strongest for fishing and swimming) when comparing to at least one of the null models. This conclusion is robust to variations in dataset size. Our study suggests that achieving the WFD water quality goals may not enhance recreational ecosystem services, and calls for further empirical research on the connection between water quality and ecosystem services.

## Introduction

Water is one of the most regulated areas in European Union (EU) environmental policy, covering topics as diverse as drinking water [1], bathing water [2], and groundwater [3]. However, early attempts to regulate Europe’s aquatic environment were characterized by serious deficits in policy implementation and effectiveness [4]. Through a combination of substantive and procedural measures the EU Water Framework Directive (WFD) [5] represents a major effort to tackle the challenges that have long frustrated endeavours of EU and national policy-makers to improve water quality in Europe. With respect to procedures, the WFD advocates, amongst others, river basin management, i.e. management activities at hydrological rather than administrative scales—the so-called River Basin Districts (RBDs)–as well as the establishment of participatory forums in water planning. The WFD thus responds to the insight that coordination problems lay at the heart of previous failures to effectively reduce water pollution in EU member states.

Over the past decades, water quality standards have evolved from unidimensional characteristics (e.g. water clarity) to multidimensional metrics that account for biological, hydro-morphological and chemical criteria [6]. For surface waters, the WFD quality assessment is based on a measurement scale that rates ecological characteristics as ‘high’, ‘good’, ‘moderate’, ‘poor’ or ‘bad’, and chemical characteristics as either ‘good’ or ‘fail’. These metrics are assessed against reference conditions before “major industrialisation, urbanisation and intensification of agriculture” [7]. For instance, ‘high’ status is characterized by the presence of “no, or only very minor, anthropogenic alterations […] from those normally associated with that type under undisturbed conditions” [8]. The overall substantive policy goal of the WFD is to achieve ‘good’ or ‘high’ overall status of both surface- and groundwaters across Europe by 2027, and to protect water bodies from further deterioration. For surface waters, good overall status is defined by high/good state in both ecological and chemical conditions.

The implementation of the WFD has been studied from various disciplinary angles and perspectives [9,10]; for example, its legal [11], ecological [12] and economical [13] implications have been addressed. However, we know little about the social benefits (ecosystem services) generated by the WFD and its outcomes. Furthermore, over the past five years, the European Commission has expressed in a number of policy documents the view that achieving ‘good’ water status will not only “allow aquatic ecosystems to recover”, but will also “deliver the ecosystem services that are necessary to support life and economic activity that depend on water” [14–17] (also see S1 Table). Yet so far, empirical evidence is scarce as to whether improved water status does actually enhance the provision and utilisation of ecosystem services [18–20].

In this paper, we test whether reaching WFD targets enhances cultural ecosystem services, specifically recreation, which is of significant economic and cultural importance in England and across Europe. Various attempts have been made to link water quality to the recreational value of inland waters (e.g. [21–26]), however, these come with a number of shortcomings. First, they typically assess the perceived value of a water body, usually with reference to economic proxies such as willingness-to-pay or the travel-cost method [21,22], rather than actual utilization. Second, the recreational value commonly comes in an aggregated form and does not distinguish between different recreational services (e.g. walking vs. swimming) that may have different water quality requirements [23,24]. Thus, few studies explicitly explore the relationship between actual indicators of water quality and a specific recreational use. As one of the few examples, Vesterinen et al. [25] found an effect of water clarity (Secchi depth) on participation in fishing, and on the frequency of fishing and/or swimming visits across a number of lakes and coasts in Finland. In a U.S. study by Ribaudo & Piper [26] total suspended sediment, total nitrogen and total phosphate had an effect on the probability that an individual went fishing but not the frequency of trips they make.

While most of the indicators in the abovementioned research are part of the composite WFD ‘overall water status’ indicator, not all WFD criteria are included. A fuller range of metrics as included in WFD water status are considered in the literature review by Vidal-Aberca et al. [27], who argue that the majority of the hydromorphological and biological indices used in WFD are likely factors in provision and use of recreational services. Nevertheless, to our knowledge only one study [19] has explicitly correlated the WFD ecological status metric to an ecosystem service: fish catch measured as catch per unit effort, in different locations along a one large boreal Finnish lake. Thus, whether the composite WFD overall water status is, as argued by European Commission official documents, an indicator that correlates with societal benefits is still unknown.

**Fig 1:**
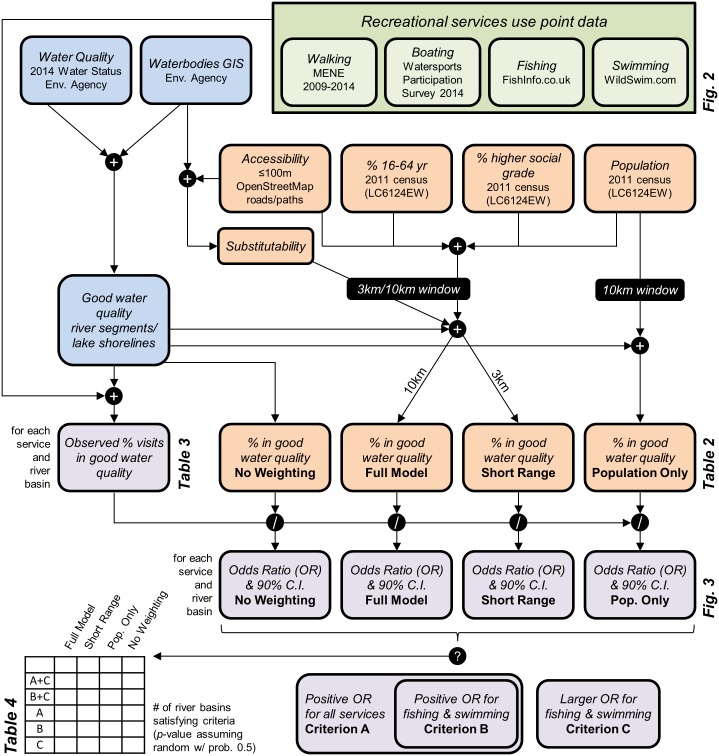
Schematic diagram of the different steps undertaken within the analysis. Multiple data sources were combined (+), compared relative to each other (/) and tested against defined criteria (?). Colors match respective Methods sections: (i) Recreation use data curation (green); (ii) Water status and geospatial data (blue); (iii) Null models (orange); (iv) Statistical analysis (purple).

To shed light on the putative association between cultural services and WFD overall water status, this paper uses data from several nationwide surveys in eight RBDs in England, which give us a unique opportunity to perform an empirical statistical analysis for different dimensions of cultural ecosystem services across a large land area. Recreation is an important ecosystem service in the United Kingdom (UK), as demonstrated by the UK National Ecosystem Assessment and its follow-on project [28,29]. Within each RBD, we use a statistical analysis comparing the frequency of four recreational activities (walking alongside water bodies, boating, fishing and swimming) in locations of good/high overall water status to different null models (see Methods and overview Fig 1). These null models account for factors such as site access, demography (population density and age distribution) and socio-economic factors (income, ethnicity or people with disability). One would expect that if good water quality is important for recreational ecosystem services there will be a positive association between WFD overall water status and locations of all or some recreational services—hereafter referred to as the ‘water quality—recreational ecosystem services hypothesis’. The association should be strongest for those services more dependent on ecological conditions that are measured/reflected by the WFD status assessment. Therefore, we also test whether the strength of association between overall good/high water status and ecosystem services is greater for fishing (which relies on fish stock) and swimming (which involves significant contact with water), and weaker for boating, and walking along rivers, canals or lakeshores (where the relationship with water is less direct).

## Methods

### Study River Basin Districts and their characteristics

Within the UK, regulation of the environment is devolved, with responsibility allocated to separate authorities for England, Northern Ireland, Scotland, and Wales, each implementing distinct policies and approaches. This results in slightly different monitoring schemes across the UK. Because of this, and the geographic limit of one of the largest datasets (MENE; Natural England’s Monitoring of Engagement with the Natural Environment), this article focuses on England and its eight RBDs only. We analysed only RBDs which are wholly within or cover large areas of England, and are under the remit of the English Environment Agency. Two further RBDs—Dee and Solway Tweed—which cover small areas of England but are principally managed by Natural Resources Wales and the Scottish Environmental Protection Agency, were excluded from the analysis.

At approximately 139,000 km in length, England’s rivers and canals, in addition to 5,700 lakes and extensive coastal, estuarine and ground waters, are a critical source of multiple and diversified ecosystem services. There are, however, dominant human activities characteristic of each basin, highlighted within the individual River Basin Management Plans [30], which may be synergistic or competitive with recreational use. The main drivers affecting water bodies across all RBDs include urbanization, agriculture, flow modification, invasive species and mining. Table 1 gives an overview of some characteristics of the RBDs and the threats impacting their water bodies, providing some context for the use of waterbodies for recreation purposes.

**Table 1:** Data obtained from individual River Basin Management Plans (Environment Agency, 2015) and Land Cover Map of Great Britain 2007 [31]. See inset in Fig 2 for location of River Basic Districts.

River Basin District	Size (km^2^)	Population (million)	% urban area	% agricultural land	% woodland	% water bodies under pressure from:						
						Physical modifications	Waste pollution	Pollution from towns, cities & transport	Changes to natural flow and level of water	Negative effects of invasive species	Pollution from rural areas	Pollution from abandoned mines
Northumbria	9,000	2.5	7	46	11	38	13	4	2	<1	10	9
North West	13,200	7	12	42	7	50	24	13	2	<1	18	3
Humber	26,100	10.8	10	65	7	42	38	16	6	<1	32	4
Anglian	27,900	7.1	5	74	6	51	50	10	10	6	47	N/A
Severn	21,000	5	6	66	10	27	29	12	7	<1	40	2
Thames	16,200	15	17	63	13	44	45	17	12	3	27	N/A
South East	10,200	3.5	7	55	13	43	40	9	7	2	30	N/A
South West	21,000	5.3	4	62	9	22	33	4	3	1	44	4

The extent of different pressures upon the waterbodies in each RBD provides some insight into the past and present uses of land and water. For example, differences in the relative importance of rural and urban/industrial activities to the local economy and the dominance of particular types of industries are key determinants in the usage of water and land. Approximately 70% of land use in England is agricultural [32], and across all eight RBDs, the majority of land is rural. The Thames RBD which includes Greater London is the most urbanised catchment, supporting the largest population and number of visitors, but the predominant economic activity—financial—does not directly utilise the river as a resource. The North West RBD similarly contains some of the most highly populated, previously industrial, urban centres in England, and its aquifers provide a crucial public water supply. However, in the North West, use of water resources is mixed as there is also a large rural economy, for which tourism to its lakes is critical. For the principally rural based economies of the Southwest and Anglian basins, water based tourism constitutes one of the main industries. This is due to the location of the Norfolk Broads (Anglian) and over half of England’s bathing waters (Southwest) within these districts.

### Recreation use data curation

**Fig 2:**
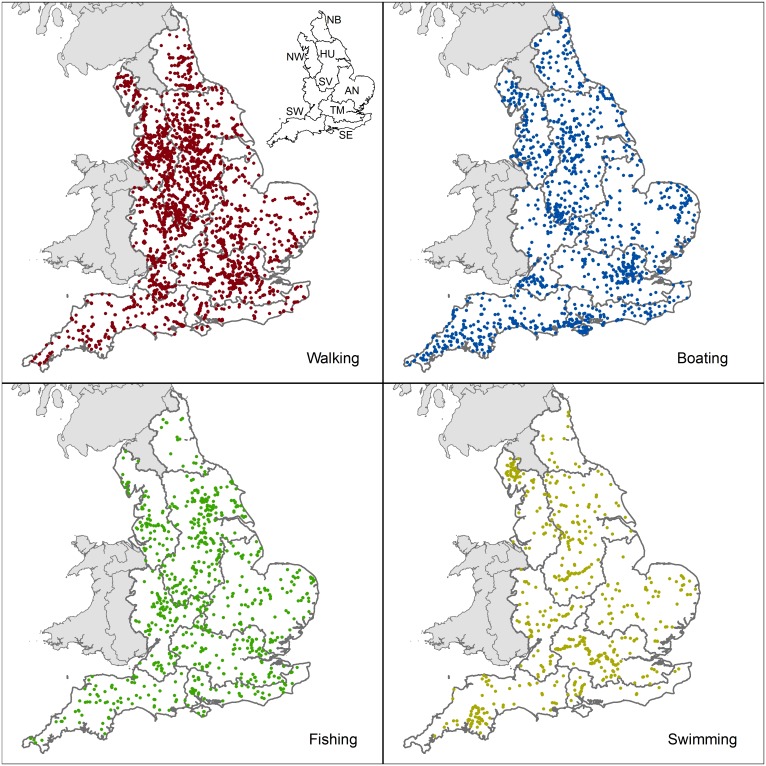
Available datasets for cultural ecosystem services use in rivers, canals and lakes across England. Geo-referenced visitation data from the Managing Engagement with the Natural Environment (MENE, Natural England 2009–2014; *n* = 4459); boating visits in the Watersports Participation Survey (British Marine, MCA, RNLI, RYA, British Canoeing and CEFAS 2014; *n* = 1298); fishing sites on fishinginfo.co.uk (Angling Trust 2015; *n* = 816); and outdoor swimming sites on wildswim.com (Outdoor Swimming Society 2015; n = 565). Inset shows the locations of the eight River Basin Districts in England (north to south): Northumbria (NB), North West (NW), Humber (HU), Anglian (AN), Severn (SV), Thames (TM), South East (SE) and South West (SW). Only points near (≤1km) of a river body with a reported ‘overall water status’ (i.e. WFD water quality standard) in 2014 were included.

We used geospatial locations of actual use of inland water (rivers, canals and freshwater lakes) for recreational services (walking, boating, fishing and swimming). Locations were obtained from nationwide surveys conducted between 2002–2014 by different agencies and an online website reporting outdoor swimming sites (Fig 2 and S1 Text).

For walking, we used data from the MENE survey [33], specifically the raw visitation data, in order to obtain locations of outdoor visits. We selected visits whose ‘visit location’ related to rivers, lakes or canals and the ‘outdoor activity’ included walking with or without a dog. For boating, we used data from the 2014 Watersports Participation Survey conducted by British Marine Federation (BMF), Royal Yachting Association (RYA), Maritime and Coastguard Agency (MCA), Royal National Lifeboat Institution (RNLI), British Canoe Union (BCU), and the Centre for Environment, Fisheries and Aquaculture Science (CEFAS) [34], from which we selected those locations where the major activity related to boating. For fishing we used a geospatial database of fisheries/venues produced by the Angling Trust [35]. We selected locations where water type was defined as river, canal or still water, excluding transitional and saltwater fisheries. Finally, for swimming we used the locations of reported swimming sites provided by the Outdoor Swimming Society [36]. Further technical details on each data source and preprocessing steps are found in the S1 Text.

To avoid issues related to uneven sampling effort across RBDs which could arise in both in-house surveys and online databases, we statistically analysed each RBD separately, and examined how many of the RBDs agree with the water quality-recreational services hypothesis. To account for uneven sampling effort within RBDs, we repeated the analysis with equal-sized subsamples for each service (see Statistical analysis).

### Water status and geospatial data

The Environment Agency (EA) reports annually on the status of individual water bodies in England based on a national standard implementation of the WFD water status classification [37]. WFD water status combines metrics on ecological integrity (e.g. fish, blooms, littoral invertebrates), physio-chemical elements (e.g. temperature, pH), geomorphology, and over 70 specific pollutants or chemicals compounds (see S1 Text for details). Some of these, for example temperature or phytoplankton blooms, can be directly sensed by people, whereas e.g. the thresholds for most chemicals are below visual and/or olfactory detection limits. As the most recently completed dataset, we used the 2014 water status classification of waterbodies, available on the EA’s website [38]. Geospatial data on the location of monitored rivers and lakes are publicly available from the EA, whereas geospatial data on the location of canals, not publicly available, were provided courtesy of the EA. All canals, rivers, and lakeshores were divided into linear segments (average length 5.3 m), resulting in about 9.5 million ‘potential sites’ for freshwater-based recreational activities. To assign water status to each record in the recreational use data, we matched the location of the visit with the nearest waterbody, keeping only those visits in our dataset which occurred within 1km of a waterbody with a valid water status. In this way we excluded water bodies whose status has not been assessed.

### Null models

According to Natural England’s MENE survey data of 2013–2014, the following factors affected the participation of people in outdoor recreation activities: age, social grade, ethnic origin, level of deprivation, and whether or not a person had a limiting illness or disability [39]. Amongst others, it was found that people over 65, in social grade DE (“Semi-skilled & unskilled manual occupations, Unemployed and lowest grade occupations”; UK Office for National Statistics (ONS)), of non-white ethnicity or with any disability are underrepresented in outdoor recreational activities. The analysis also shows that 68% of all visits were within 2 miles (3.2 km) and 83% of all visits within 5 miles (8 km) of a respondent’s home. Using the MENE data, Bateman et al. [40] similarly found that income, percentage of retired people, percentage of non-white ethnicity, total population and travel time to be highly statistically significant, in addition to variables reflecting land cover and substitutions within a 10 km radius. In contrast to the MENE analysis, however, the effect of proportion of retired people was positive rather than negative. Neither analysis focused specifically on water nor considered the importance of water status in people’s choice of recreation sites. Narrowing MENE (and the other datasets) to include only locations nearby water bodies limits the applicability of the approach used by Bateman et al. which requires very large sample sizes. Instead, we developed a null model of the predicted percentage of visits to good/high status sites within a RBD, and compared that with actual use data. We developed four variants of this null model (Table 2), variously including the effect of demand (population, age, income/social grade), substitutability (alternative options within short travel distance from home), and accessibility (distance to OSM road layer features). The four variants test the sensitivity and robustness of our results to null model assumptions.

**Table 2:** Null models for the expected % of visits in good/high overall water status sites.

Null model	Description	Expected % in Good/High Overall Water Status (*e*_j,good_)							
		Northumbria	North West	Humber	Anglian	Severn	Thames	South East	South West
Full	Weight each river body segment by the ratio of demand and substitutability. Demand is calculated as a 10km radius aggregated population density of adults (age 16–64) with higher income (excluding social grade DE). Substitutability is a 10km (proximity to home) aggregated linear kilometres of rivers, canals and lakeshores. To account for public accessibility, only river/canal/lakeshore segments closer than 100m of a road/path/trail in Open Street Map are included	11.9%	21.7%	17.5%	12.1%	17.9%	3.9%	14.7%	17.3%
Short Range	Same as ‘Full’ model but assuming shorter trips, with a 3.3km radius (proximity to home) buffer around each river body segment	15.6%	17.4%	15%	9.7%	15.2%	2.5%	13.4%	16.7%
Population Only	Weighting based on 10km radius aggregated population density, includes all river body segments regardless of accessibility	11%	22.1%	24.3%	11.1%	20.6%	4.4%	15.6%	17.3%
No Weighting	All river body segments included with equal probability	27.9%	28.3%	15.1%	10.2%	17.2%	9.5%	12%	18.9%

The general form of the null model for the percentage of visits to locations withgood/high water status in RBD *j* is

$$e_{j,good}=\frac{\sum_{i\in S\left(j\right)}w_{i}\;g_{i}}{\sum_{i\in S\left(j\right)}w_{i}}$$

where g_i_ = 1 for potential sites *i* within the RBD (S(*j*)) where overall water status was classified as ‘good’ or ‘high’. Variants of the model were created using different weighting functions *w*_*i*_−*w*_*i*_ = 1(‘No Weighting’), $w_{i}=\sum_{k\in A_{10}\left(i\right)}p_{k}$ where A_x_(*i*) is a radius of 10 km around site *i* and *p*_k_ the population density in pixel *k* on a 100m resolution map of the UK (‘Population Only’), and $w_{i}=\sum_{k\in A_{x}\left(i\right)}r_{k}p_{k}a_{k}i_{k}/\sum_{k\in A_{x}\left(i\right)}r_{k}l_{k}$ where *a*_*k*_ is the percent of population between 16–65 years of age, *i*_*k*_ is the percentage of working age people not in social grade DE, *r*_*k*_ = 1 if a site is within 100m from a road/pathway and zero otherwise and *l*_*k*_ is the sum of linear length of accessible rivers, canals and lakeshores within pixel *k* (‘Full’ model *x* = 10; ‘Short Range’ model *x* = 3.3).

Spatial data for the null models were processed as follows (see also Fig 1). To proxy demand, UK census population data of 2011 [41] were converted and gridded at 100m resolution to create a map of population density. In the ‘Full’ and ‘Short Range’ models, this was further filtered by age (including only population aged 16–64 years) and social grade (namely excluding social grade DE) based on UK census data [42]. Social grade is a system of demographic classification in the UK, ranging from upper middle class (A), middle class (B), lower middle class (C1), skilled lower middle class (C2), working class (D), and non-working (E). To analyse the accessibility of sites we used the Open Street Map (OSM) road layer [43], which displays roads, footpaths, and bridleways. We define accessibility as the distance to the nearest feature in the OSM road layer. Nearly 90% of visit data describes locations within a distance of 100m from OSM road layer features (S1 Fig). Unfortunately, information related to public access rights were not consistently available for all roads. We therefore assumed that all OSM features are publically accessible, and so is any river, canal or lakeshore stretch within 100m of these. Substitutability was defined as the total linear length of accessible water bodies (i.e. potential recreational sites) in the vicinity of a site. As a simple proxy for travel time and travel distance, we performed a spatial integration of substitutability and demand over a radius of 10 km (or 3.3 km in the ‘Short Range’ null model).

### Statistical analysis

Our analysis relies on the Odds Ratio (*OR*), contrasting the odds that a member of a specified population will fall into a certain category with the odds that a member of another population will fall into the same category. To this end, we distinguished visits to sites with good/high water status and visits to sites characterized by moderate/poor/bad status. We then compared the actual visitation data to data derived from random sampling, based on the null models described above, for all potential recreation sites. Formally, *OR*_*j*_ = *n*_*j*,*good*_/(*n*_*j*_ − *n*_*j*,*good*_)·(1 − e_*j*,*good*_)/e_*j*,*good*_ where *n*_*j*,*good*_ is the total number of visits to water bodies with a good/high status in RBD *j*. An *OR*_*j*_ value larger than 1 means positive association, namely that recreational activities are more likely to take place in locations with good/high overall water status. Following Grissom & Kim [44], we calculated a 90% confidence interval, assuming the natural logarithm of *OR* is normally distributed, or *ln*(*OR*) ± *z*_*α/*2_*S*_*ln*(*OR*)_ where *z*_*α/*2_ = 1.645 is the value of a two-sided standard normal distribution at 90 per cent, and *S*_*ln*(*OR*)_^2^≃(*n*_*j*,*good*_ + 0.5)^−1^(*n*_*j*_ − *n*_*j*,*good*_ + 0.5)^−1^ where we neglect the terms arising from the much larger sample of population 2 and use the standard bias correction constant [46]. We independently calculate *OR*_*j*,*i*_ for each of the four recreational services (*i* = *walk*, *boat*, *fish*, *swim*) in each RBD *j*.

To test the ‘water quality—recreational ecosystem services’ hypothesis, we considered three quantitative criteria. First (a), we expect all recreational services to have a positive association with WFD overall water status (*OR*_*j*,i_ > 1). Secondly (b), if the former does not hold, we at least expect that both swimming and fishing—services with direct and prolonged contact with water—would show positive association (*min*(*OR*_*j*,swim_,*OR*_*j*,fish_) > 1). Finally (c), we expect walking and boating to have weaker association than swimming and fishing, where *max*(*OR*_*j*,walk_, *OR*_*j*,boat_) < *min*(*OR*_*j*,swim_,*OR*_*j*,fish_). Expecting that at least 50% of the RBDs would agree to criteria if the hypothesis is true, we calculated *p*-values based on binomial probabilities to observe an equal or smaller number of RBDs meeting the criteria by random. To test if the different *n* for the four datasets affected our results, we repeated this analysis with randomly sub-sampled datasets for walking, boating and fishing with same *n* as swimming (see S1 Text).

## Results

The location and number of site visits related to four ecosystem services—walking, boating, fishing and swimming—as determined by the four surveys used, comprised of a total of 7,177 data points (Table 3). According to these data sets, 22.8% of all walking (alongside a waterbody), 17.9% of all boating, 13.7% of all fishing, and 15.7% of all swimming visits in England took place at sites classified as good/high water status. However, we observe a great degree of variation between the eight RBDs in England. For example, the percentage of walking visits made to good/high water status sites ranges from 5.7% in Anglia to 47.9% in the North-West (Table 3). Likewise, few visits (for all activities) in the Thames RBD take place in sites characterized by a good water status (2.6 to 7%), whilst users in Northumbria and the North-West recreate more often at sites with good/high water status (17.9 to 47.9%).

**Table 3:** Number of visits and percent in good/high overall water status sites in all eight River Basin Districts.

Dataset	Survey (Year/s)	Source of Data	Criteria for inclusion	*n*_j_ (% in Good/High Overall Water Status = *n*_j,good_ / *n*_j_)							
				Northumbria	North West	Humber	Anglian	Severn	Thames	South East	South West
Walking	MENE (2009–14)	Natural England	*Question 5* option 4 (“Specific visit location included—River, Lake or Canal”) positive & *Question 4* option 15 (Walking Without a Dog) and/or option 16 (Walking With a Dog) positive	186 (20.4%)	624 (47.9%)	1467 (24.5%)	597 (5.7%)	657 (23.7%)	527 (7.0%)	138 (25.4%)	282 (22.3%)
Boating	Watersports Participation Survey (2014)	British Marine	*Activity* marked “total boating visits”	71 (25.4%)	175 (28.0%)	273 (16.8%)	160 (9.4%)	105 (23.8%)	182 (6.0%)	121 (14.9%)	229 (21.4%)
Fishing	FishingInfo.co.uk (accessed 8/15)	Angling Trust	*Water type* is river, canal or stillwater	17 (35.3%)	78 (17.9%)	244 (13.1%)	115 (10.4%)	115 (14.8%)	116 (3.4%)	58 (8.6%)	74 (21.6%)
Swimming	WildSwim.com (accessed 8/15)	Outdoor Swimming Society	*Site type* is river (include canals) or lake	14 (42.9%)	74 (18.9%)	118 (9.3%)	61 (19.7%)	62 (11.3%)	117 (2.6%)	25 (32.0%)	95 (23.2%)

Expected frequencies of visits to sites with good/high water quality, as predicted by the null models, similarly differ between the river basins but, to an extent, are also dependent upon which null model is applied (Table 2). According to the basic ‘No Weighting’ model, expected visits to sites with good/high status range from 9.5 to 27.9% across all eight RBDs. However, incorporating population density, household income, substitutability, accessibility and proximity to home (within a 10km radius) substantially changes these rates. Most notably, expected visits to good/high status sites in Northumbria decreased from 27.9 to 11.9%, in the North West from 28.3 to 21.7%, and in the Thames RBD from 9.5 to 3.9% (‘No Weighting’ model compared to the ‘Full’ model, Table 2). Assuming a shorter travel distance (3.3 km radius), by applying the ‘Short Range’ model, only slightly reduces expected rate of good/high status site visits when compared to the ‘Full’ model. Furthermore, there were no notable differences between the ‘Population Only’ model and the ‘Full’ model (Table 2). All null models predict that the rate of visits to good/high water status sites is lowest in the Thames RBD and generally high (>15%) in the North West, Humber, Severn, and South West RBDs.

**Fig 3:**
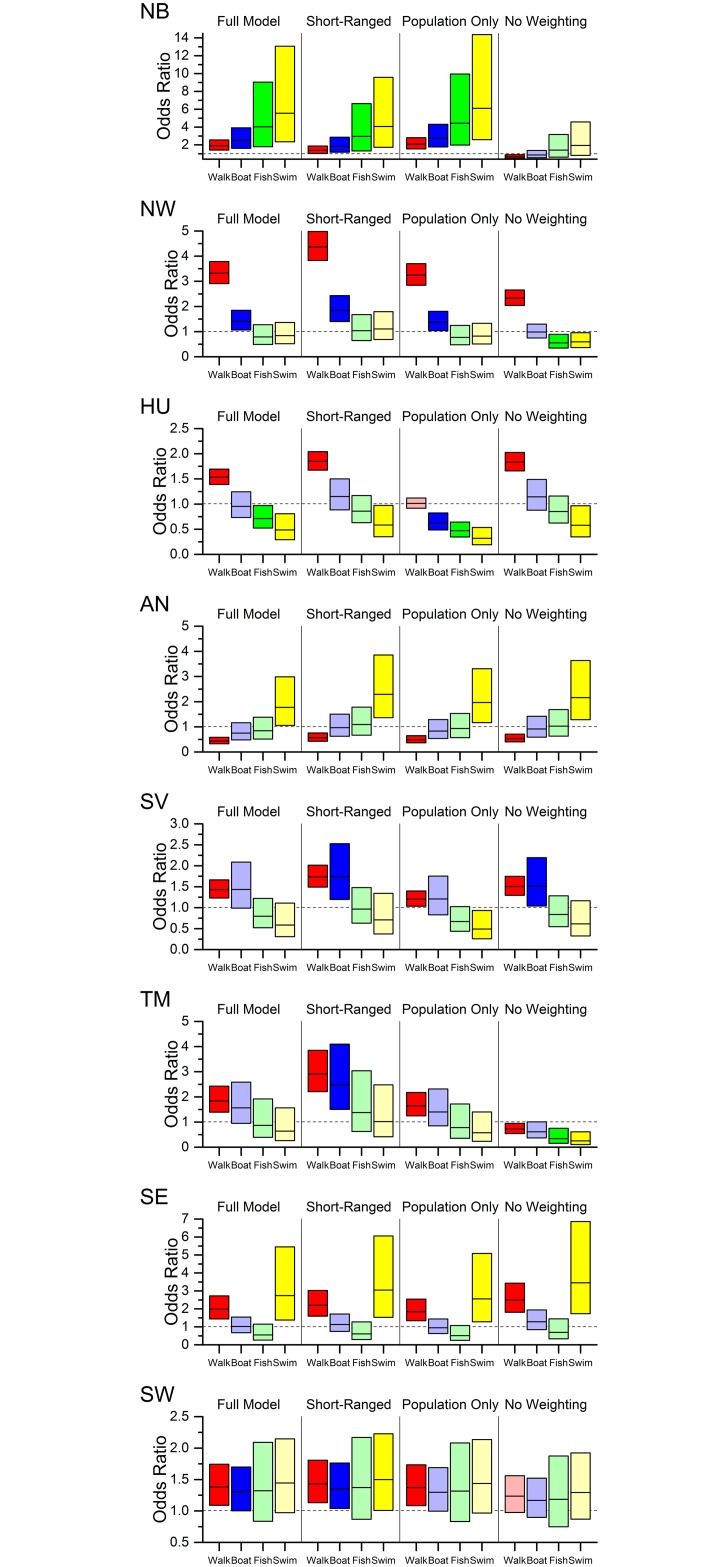
Association of good/high overall water status and use of cultural ecosystem services for the eight River Basin Districts. The Odds Ratio (*OR*) of each River Basin District measures the likelihood that actual visits take place in sites characterized by good/high overall water status compared to random locations selected under a null model accounting for demand and substitutability (Table 2). *OR* exhibits a statistically significant positive (negative) association (i.e. visits in good/high overall water status sites are more (less) common than random; solid colours) if the 90% confidence interval is completely above (below) the line *OR* = 1. The robustness of the results is tested by comparing null models, including a null model without weighting. See Fig 2 for River Basin Districts acronyms.

The Odds Ratio (*OR*) analysis showed that the actual number of visits by walkers to good/high status sites is higher than expected (under weighted null models) in seven RBDs, and significantly so in most (Fig 3, red boxes). For boating, the ‘Full’ model suggests higher probabilities of visits to good/high status sites in only five RBDs, with the difference significant in only three (Northumbria, North West, South West; blue boxes in Fig 3). Using the ‘Short Range’ model, the other two positive associations (Severn and Thames RBDs) become significant, while in the Humber RBD the ‘Population Only’ model shows a significant negative association. Across all null models, fishing is positively linked to water status in Northumbria and the South West, but negatively associated in the Humber, Severn and South East (Fig 3, green boxes). However, significant associations are only generated by some models in Northumbria and the Humber. Finally, under the ‘Full’ model, swimming visits are positively associated with water status in four RBDs (significantly so in three) but negatively associated in the other four RBDs (significantly so in only the Humber; yellow boxes in Fig 3). In the ‘Short Ranged’ model, the association between water status and swimming visits is significantly positive in one additional RBD (South West). In the ‘Population Only’ and ‘No Weighting’ models, the correlation between water status and swimming visits is significantly negative in up to three additional RBDs.

We test the ‘water quality—recreational ecosystem services’ hypothesis by examining the number of RBDs agreeing to different quantitative criteria (see Methods). Postulating that the hypothesis implies positive association of water quality with all services, and stronger association for swimming and fishing, we find that at most one RBD (Northumbria; NB) agrees to both criteria (‘(a)+(c)’; Table 4). Even if one expects as few as 50% of RBDs tested to agree to all criteria (assuming the hypothesis is true), this result is highly unlikely by chance alone (*p* < 0.05 based on a binomial distribution). Relaxing the criteria, demanding only swimming and fishing are positively associated with status (‘(b)+(c)’) we get either 1 or 2 RBDs agreeing to both criteria (*p* between 0.035 and 0.145) which is still unlikely. Further relaxing those criteria, and using a null model which favours shorter trips (‘Short-Ranged’ model) would gradually increase the number of RBDs that match. Still, in 17 of 20 combinations of Table 4, the probability to observe the same or fewer RBDs in agreement with results is less than 15%. If one increases the expected probability from 50% to 60% (or more) we find that 17 (or more) of these 20 combinations have a *p* < 0.05. Demanding the positive associations are statistically significant (i.e. 90% C.I. is above *OR* = 1), we get only one RBD (Northumbria) (*p* = 0.035) that meets the criteria (a, b and c and their combinations as in Table 4) for the ‘Full’, ‘Short-Ranged’ and ‘Population-Only’ models, and none for the ‘No Weighting’ model.

**Table 4:** Number (and codes) of River Basin Districts agreeing with the water quality—recreational ecosystem services hypothesis (or variants thereof): if good water quality is important for recreational ecosystem services we expect either (a) a positive association with water quality for all services or (b) positive association at least for services with significant direct contact with water—swimming and fishing, (c) stronger association with water quality for swimming and fishing relative to walking and boating. The *p*-values denote the probability of getting equal or fewer RBDs meeting the criteria by chance alone, assuming a binomial distribution with 0.5 probability of success per trial.

Criteria held	Full Model	Short-Ranged	Population Only	No Weighting
(a)+(c)	1* (*p* = 0.035) (NB)	1* (*p* = 0.035) (NB)	1* (*p* = 0.035) (NB)	0* (*p* = 0.004)
(b)+(c)	1* (*p* = 0.035) (NB)	2 (*p* = 0.145) (NB, AN)	1* (*p* = 0.035) (NB)	2 (*p* = 0.145) (NB, AN)
(a) only	2 (*p* = 0.145) (NB, SW)	4 (*p* = 0.637) (NB, NW, TM, SW)	2 (*p* = 0.145) (NB, SW)	1* (*p* = 0.035) (SW)
(b) only	2 (*p* = 0.145) (NB, SW)	5 (*p* = 0.855) (NB, NW, AN, TM, SW)	2 (*p* = 0.145) (NB, SW)	3 (*p* = 0.363) (NB, AN, SW)
(c) only	2 (*p* = 0.145) (NB, AN)	2 (*p* = 0.145) (NB, AN)	2 (*p* = 0.145) (NB, AN)	2 (*p* = 0.145) (NB, AN)

To ensure these results are not affected by differences in sampling between services, we performed similar analysis with randomly sub-sampled visitation data for walking, boating and fishing (S2 Table) with similar *n* to those of swimming. We find that between 0.7±0.5 (mean and standard deviation for ‘Full’ model for ‘(a)+(c)’ criteria) RBDs to 1.3±0.8 RBDs (‘Short-Ranged’ model, ‘(b)+(c)’ criteria) conform with the more stringent sets of criteria of the water quality-recreational ecosystem services hypothesis. Furthermore, in 16 of 20 combinations of S2 Table, we get at most 2 RBDs (*p* < 0.15) agreeing with criteria for 9 or more of 10 random realizations. These results are similar to results based on the full datasets.

## Discussion

Our results do not support our original 'water quality—recreational ecosystem services hypothesis' that there would be a consistent positive association between WFD water status and service utilization. Moreover, of all four recreational ecosystem services, walking is most consistently and strongly associated with good/high water status. In other words, the association is strongest for the activity with the least direct relationship with water. In testing our hypothesis, we controlled for a variety of socio-economic and geographical factors that could also affect site choice, such as population density, age, ethnic characteristics, income, substitutability of sites, and site access. The results held, even when controlling for different null models, quantitative criteria, and dataset sizes. We offer four possible explanations for these somewhat counter-intuitive findings.

First, water status as defined by the WFD may not adequately reflect water quality priorities of the wider public. For example, the biodiversity of aquatic invertebrates, one of the WFD metrics, may be irrelevant for swimmers and boaters. The presence of litter or debris, in contrast, might discourage water use although it has little impact on the status of a water body [45–49]. Water temperature contributes to status assessments, but reference conditions may be cooler than those preferred by swimmers [50]. Water users seem to prefer clearer waters [25,47,48], but good ecological status may be associated with relatively poor clarity in certain water body types, for instance in humic inland lakes [25].

Second, the relationship between water quality and cultural ecosystem services may be non-linear [51]. For example, the difference between poor and medium water status may be more noticeable than the difference between medium and good/high status. In other words, although achievement of good overall water status is the objective of the WFD, this transition may not be the most critical for recreational site use.

Third, WFD water status may simply be unimportant when making site choices—or at least much less important than other factors. In our null models we controlled for a number of socio-economic and geographical factors, but possibly missed some important local effects. To illustrate, a water body must meet certain fundamental criteria to facilitate recreational use: sufficiently large to launch a boat, reasonably safe for swimmers, or with relevant permissions for angling. Furthermore, some ecosystem services require specific types of infrastructure, for instance boating ramps or convenient swimming access points. Natural resource management decisions (e.g. fish stocking) and strategies related to the touristic marketing of sites may further affect site choice. Finally, site choice could be driven by non-water environmental characteristics such as surrounding land use [40], naturalness/wildness, presence or absence of shade, and wind [50]. Together, these infrastructure and management factors may limit site choice, meaning water status must be compromised in favour of practicality.

Fourth, it could be hypothesized that the status of waters in England has improved quickly in the more recent past. Society, however, has a long ‘memory’ for preferred recreational sites. People thus keep visiting places with potentially poorer water quality because locations with good/high water status have not yet been ‘discovered’ or become well known. The plausibility of this argument, however, is undermined by the fact that, according to the EA, water has not improved significantly between 2008 and 2012 (S3 Table). However, given the actual implementation of the WFD in the UK is very recent, with the first round of River Basin Management Plans published in 2009, it is possible some new measures may still impact recreational use in the future.

Nevertheless, we found, across all null models and in all but one RBD, a remarkably consistent association between water status and walking visits (Fig 3). Walkers may be more responsive to water status (since they are less restricted to specific water bodies by factors such as hydromorphology and infrastructure), and not as influenced by inter-service competition as are boaters, swimmers or fishers. Furthermore, they have the option of walking in other ‘green spaces’, not along water bodies, so may be more selective as to the water quality when choosing ‘blue space’ recreation sites.

Our data also highlight regional differences in the association between water status and recreational use (Fig 3). Most notably, Northumbria and the South West are the only RBDs in which all activities are positively related to water status (when compared to the weighted null models). In most RBDs we find a pattern of decreasing association from walking-boating-fishing-swimming, but in the Northumbrian and Anglian RBDs this pattern is reversed. Detailed exploration of these regional differences is beyond the scope of this paper, but as potential explanations we suggest regional idiosyncrasies in (i) demography, with younger people being more critical of water quality [49], (ii) frequency of recreational water use, with more frequent water users more sensitive to differences in water status [52], (iii) relative importance of site choice factors, in that residents of more urbanized RBDs may be less sensitive to water status, (iv) differences in typical travel distances (willingness to travel farther) for different populations in different regions of England, (v) attachment to specific sites irrespective of water quality, through force of habit [53,54] or marketing of specific sites [55].

## Conclusion

Using the case of England our analysis shows no, or even negative, correlation between WFD water status and spatial patterns of recreational services, in particular fishing and swimming. This undermines recent arguments about the benefits of the WFD, and warns that achieving ‘good’ or ‘high’ overall water status may not, in fact, improve the provision and utilization of ecosystem services. Extending the analysis to other parts of the UK and Europe, perhaps using citizen science approaches to collect recreational use data [56], is necessary to validate the generality of our findings and explore the spatial variation across RBDs. Further research should also explore if the relationship between water quality and recreational services is different in developing countries, where water quality is generally poorer than in present-day Europe. Necessary datasets (see schematic Fig 1) may possibly include a combination of crowdsourced water quality data (e.g. E. coli crowdsourced testing in India [57]), social-media (e.g. Flickr) for recreational use data, and emerging global datasets (e.g. world population [58], remote-sensed poverty map [59]).

The ecological integrity of Europe’s aquatic ecosystems is threatened by a range of anthropogenic and natural pressures. This article suggests that if the aim of water legislation in the EU is to maintain the services these waters provide to society, it is necessary to improve the WFD monitoring system to capture other dimensions affecting supply and demand, especially of cultural services. This will necessitate involving also social scientists and the public in defining metrics and targets, not only freshwater ecologists and ecotoxicologists, to form a truly trans-disciplinary water framework for Europe.

## Supporting Information

S1 Fig Cumulative distribution for the distance of individual visits from Open Street Map road/trail/path. The vast majority of visits are near a road/trail/path which demonstrates a strong dependence of these cultural ecosystem services on accessibility. The percent of visits within 100m of a road/trail/path is 92.5% for walking (red), 92.9% for boating (blue), 60.9% for fishing (green), 80.0% for swimming and 87.9% in the combined dataset comprising all data (black dotted).
(DOCX)

S1 Table Ecosystem services as an integral part of the WFD: evidence in official documents. (DOCX)

S2 Table Number of River Basin Districts agreeing with the water quality—recreational ecosystem services hypothesis (or variants thereof) with sub-sampling of Walking (12.67% of available data), Boating (43.52%) and Fishing (69.24%) to match Swimming *n*. Values are averages and standard deviations of the number of RBDs agreeing with criteria in 10 random boot-strapping realizations, and percentage of realizations with 2 or less RBDs (*p* < 0.15) matching criteria.
(DOCX)

S3 Table Status classifications of UK surface water bodies in percent (including Wales and Scotland) under the WFD. (DOCX)

S1 Text Supporting Information. (DOCX)
