# Supplementary material for: Water Quality Is a Poor Predictor of Recreational Hotspots in England
Source: PLoS One. 2016 Nov 22;11(11):e0166950. doi: 10.1371/journal.pone.0166950 (PMC5119820; doi:10.1371/journal.pone.0166950)

# S1 Fig. Cumulative distribution for the distance of individual visits from Open Street Map road/trail/path

The vast majority of visits are near a road/trail/path which demonstrates a strong dependence of these cultural ecosystem services on accessibility. The percent of visits within 100m of a road/trail/path is 92.5% for walking (red), 92.9% for boating (blue), 60.9% for fishing (green), 80.0% for swimming and 87.9% in the combined dataset comprising all data (black dotted).


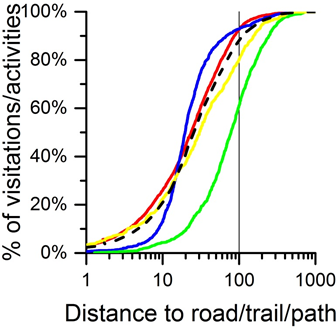

Supplement: S1 Fig — The vast majority of visits are near a road/trail/path which demonstrates a strong dependence of these cultural ecosystem services on accessibility. The percent of visits within 100m of a road/trail/path is 92.5% for walking (red), 92.9% for boating (blue), 60.9% for fishing (green), 80.0% for swimming and 87.9% in the combined dataset comprising all data (black dotted). (DOCX) [file pone.0166950.s001.docx]
