# Supplementary material for: Water Quality Is a Poor Predictor of Recreational Hotspots in England
Source: PLoS One. 2016 Nov 22;11(11):e0166950. doi: 10.1371/journal.pone.0166950 (PMC5119820; doi:10.1371/journal.pone.0166950)
Supplement: S1 Table — (DOCX) [file pone.0166950.s002.docx]

# S1 Table. Ecosystem services as an integral part of the WFD: evidence in official documents

| **Excerpt** (emphasis: authors) | **Reference** |
| --- | --- |
| Section 5.1: The **WFD objective of good status** is necessary to ensure long term availability of sufficient water of good quality. Achieving good status for all waters will allow aquatic ecosystems to recover and **to** **deliver the ecosystem services** that are necessary to support life and economic activity that depend on water. | [10](#_ENREF_10) |
| Section 5.6: The assessment of the RMBPs shows the poor quality of the assessment of costs and benefits. A strong improvement in this area and the definition of a shared methodology for the calculation of costs (including environmental and resource costs) and **benefits (including ecosystem services)** is necessary. Otherwise, it will be possible neither to ensure the implementation of effective pricing policies nor to avoid disproportionate and inadequate measures. |  |
| Section 2.2: Greater consideration is now being given to the **importance of protecting ecosystem services**. The Water Framework Directive specifically includes certain ecosystem service provisions (on drinking water source protection and on other protected areas). However, it is true that its primary focus is on the state of the biological, chemical and hydromorphological character of water bodies, rather than on the services they provide (which would require a definition of such services). This focus is important in order to deliver important objectives for ecosystem health, such as enhancing Europe’s biodiversity resource and contributing to the EU Biodiversity Strategy.  In order to ensure that the notion of good status under the Directive continues to meet its **objective of ensuring the integrity of the aquatic ecosystems and their capacity to maintain their services**, more focus should be given to these concepts, both within the framework of the CIS process and also via other policies so that they can be better reflected in the implementation on the ground | [11](#_ENREF_11) |
| Section 2.5: The Blueprint strives to achieve widespread improvement in aquatic ecosystems, which will contribute positively to the EU Biodiversity Strategy goal of **halting the loss of biodiversity and the degradation of ecosystem services** in the EU by 2020, and restoring them in so far as feasible | [12](#_ENREF_12) |
| Section 2.6.1: The environment as a whole is affected by the state of waters. Indeed, there is a bidirectional relationship between the status of water bodies and the health of ecosystems: The **status of surface water** and groundwater bodies affects the state of aquatic and terrestrial dependent ecosystems which **greatly impacts their functions and their capacity to provide ecosystem services**. | [13](#_ENREF_13) |
| Section 6.4.3: The **improvement of the state of water resources** will trigger substantial economic benefits in terms of more efficient allocation of resources, reduction of cross-sectoral externalities (cost of de-pollution) and **provision of ecosystem services** and reduction of damages from extreme events |  |
