# Supplementary material for: Water Quality Is a Poor Predictor of Recreational Hotspots in England
Source: PLoS One. 2016 Nov 22;11(11):e0166950. doi: 10.1371/journal.pone.0166950 (PMC5119820; doi:10.1371/journal.pone.0166950)
Supplement: S2 Table — Values are averages and standard deviations of the number of RBDs agreeing with criteria in 10 random boot-strapping realizations, and percentage of realizations with 2 or less RBDs (p < 0.15) matching criteria. (DOCX) [file pone.0166950.s003.docx]

# S2 Table. Number of River Basin Districts agreeing with the water quality - recreational ecosystem services hypothesis (or variants thereof)

with sub-sampling of Walking (12.67% of available data), Boating (43.52%) and Fishing (69.24%) to match Swimming *n*. Values are averages and standard deviations of the number of RBDs agreeing with criteria in 10 random boot-strapping realizations, and percentage of realizations with ≤2 RBDs (*p* < 0.15) matching criteria.

| **Criteria held** | **Full Model** | **Short-Ranged** | **Population Only** | **No Weighting** |
| --- | --- | --- | --- | --- |
| (a)+(c) | 0.7±0.5 (100%) | 0.7±0.7 (100%) | 0.7±0.5 (100%) | 0±0 (100%) |
| (b)+(c) | 0.9±0.6 (100%) | 1.3±0.8 (100%) | 1.1±0.7 (100%) | 1.1±0.7 (100%) |
| (a) only | 1.9±0.3 (100%) | 3.4±0.5 (0%) | 1.9±0.3 (100%) | 0.7±0.5 (100%) |
| (b) only | 2.1±0.3 (90%) | 4±0.7 (0%) | 2.3±0.5 (70%) | 2.2±0.8 (60%) |
| (c) only | 1.7±0.7 (90%) | 1.7±0.7 (90%) | 1.7±0.7 (90%) | 1.7±0.7 (90%) |
