# Supplementary material for: Water Quality Is a Poor Predictor of Recreational Hotspots in England
Source: PLoS One. 2016 Nov 22;11(11):e0166950. doi: 10.1371/journal.pone.0166950 (PMC5119820; doi:10.1371/journal.pone.0166950)
Supplement: S3 Table — (DOCX) [file pone.0166950.s004.docx]

# S3 Table. Status classifications of UK surface water bodies in percent (including Wales and Scotland) under the WFD

|  | **Percent of River Bodies by Water Status*** | | | | |
| --- | --- | --- | --- | --- | --- |
|  | **2008** | **2009** | **2010** | **2011** | **2012** |
| Rivers and canals | | | | | |
| High | 2% | 2% | 2% | 2% | 2% |
| Good | 30% | 30% | 31% | 31% | 31% |
| Moderate | 50% | 49% | 47% | 47% | 46% |
| Poor | 14% | 15% | 17% | 17% | 17% |
| Bad | 4% | 4% | 4% | 4% | 4% |
| Lakes | | | | | |
| High | 6% | 5% | 5% | 6% | 6% |
| Good | 37% | 38% | 38% | 32% | 31% |
| Moderate | 44% | 44% | 44% | 46% | 46% |
| Poor | 10% | 10% | 11% | 14% | 14% |
| Bad | 3% | 3% | 2% | 2% | 2% |

* Source: Joint Nature Conservation Committee (2015) Surface Water Status (downloadable at http://jncc.defra.gov.uk/page-4250)
