# Supplementary material for: Water Quality Is a Poor Predictor of Recreational Hotspots in England
Source: PLoS One. 2016 Nov 22;11(11):e0166950. doi: 10.1371/journal.pone.0166950 (PMC5119820; doi:10.1371/journal.pone.0166950)
Supplement: S1 Text — (DOCX) [file pone.0166950.s005.docx]

# S1 Text. Supporting Information

## Classification of surface water bodies to ‘good’ or ‘high’ overall water status

The WFD classification uses the ‘one out all out’ approach, meaning that the lowest indicator value is picked out when describing the whole water body. This ensures the WFD status is highly conservative. A range of biological and chemical indicators are used in the WFD to provide an indication of either biological or chemical status of the water body. The overall organization of the WFD classification is outlined in the figure below [38]:

In all water bodies the taxonomic composition and abundance of macrophytes, phytobenthos, invertebrates, fish and phytoplankton are all taken as biological indicators. Within the high/good WFD status framework slight reductions in these measures, in comparison to the type-communities under undisturbed (pristine) conditions, are permitted. Ratios of disturbance-sensitive and non-sensitive species are also taken into account for invertebrates, macroalgae and angiosperms. A decrease in disturbance-sensitive species and overall abundance would indicate undesirable conditions that were likely a result of anthropogenic action. High/good status water bodies would have only slightly reduced species abundance and little change in the sensitive non-sensitive species ratio. Disturbance is further measured in fish populations though the age structure of the community. Missing age groups could indicate disturbance of the species or unfavourable conditions hindering reproduction or development. In order to classify as a high/good water body only slight signs of distortion in the community age structure are permitted. Phytoplankton biomass and growth rate is also monitored. An increase in growth rate and/or biomass would suggest unfavourable conditions for other species groups. Finally, disturbance in measured through the coverage of macroalgae and angiosperms. A greater level of disturbance in macroalgae and angiosperms is assumed to correlate with increased disturbance to the wider community therefore resulting in unfavourable conditions. In accordance with the other biological measures, only slight variations in these measures are permitted in water bodies of high/good status. Detailed guidelines and numeric cut-offs for the different criteria, including maximally allowed concentrations of ‘priority (hazardous) substances’ (which determines chemical status), have been developed by each Member State as part of the adoption process of the WFD. For England and Wales, this information is available in [37].

## Sourcing of Cultural Ecosystem Services use data for England

This study uses four national-level datasets on recreational service use across England. Below we detail the key technical specifications of each dataset as well as further considerations related to sampling and the spatial stratification of responses.

Monitor of Engagement with the Natural Environment (MENE):

This survey collects information about the ways that people engage with their natural environment such as visiting the countryside, enjoying green spaces in towns and cities, watching wildlife and volunteering to help protect the natural environment. The data collected includes the type of destination and main activities pursued. Each week, in-home structured interviews are undertaken with a representative sample of the English adult population (aged 16 and over) with a minimum sample of 800 across 100 sample points. The survey records information regarding all visits of the interviewee in the 7 days prior to interview, one trip is then selected at random by the Computer Assisted Personal Interviewing Software, and the location and activities carried out during that trip recorded. Less than half of respondents for each survey year visited the natural environment in the 7 days prior to survey and thus information on a selected trip was recorded for a lower sample size. Multi-stage stratified sampling is replicated for each sequential wave of survey fieldwork using the TNS in-house Omnibus Survey. This is a computerised sampling system which integrates data from the Post-Office Address file with UK 2001 Census small area data at output area level. This strategy maximises accuracy of address selection, statistical accuracy of the sampling and geographical dispersion of the samples [39].

### Watersports Participation Survey:

The Watersports Participation Survey has, since 2002, been conducted on an annual for the British Marine Federation in conjunction with other organizations with a remit in water-based recreation. In 2014, the survey partners were the British Marine Federation (BMF), Royal Yachting Association (RYA), Maritime and Coastguard Agency (MCA), Royal National Lifeboat Institution (RNLI), British Canoe Union (BCU), and the Centre for Environment, Fisheries and Aquaculture Science (CEFAS) [34] This survey records postcode level participation in twelve different boating activities (canal boating, canoeing/kayaking, motor boating/cruising, power boating, rowing/sculling, small sail boat activities - dinghies, small sail boat racing - dinghies, personal watercraft - jet-ski/similar, water skiing/wake-boarding, windsurfing, yacht cruising, yacht racing) and other blue-space recreational activities (e.g. spending leisure time on beach). In 2014, a bespoke online survey was added in which 2,562 participants pinpointed the location of their last water-based activity; these precise data were used in the present study.

### Angling Trust’s fishinginfo.co.uk Database:

Together with the Environment Agency, the Meteorological Office and Post Office, the Angling Trust developed a website (fishinginfo.co.uk) and a mobile app with a view to providing anglers with up-to-date information on the location of good fishing sites across England and Wales. The purpose of this website is to promote recreational fishing. We therefore assume that any bias in the locations within the database would tend towards better quality fishing sites. Anglers can also submit additional or corrected information, which may create a selection bias towards better sites. In view of the non-significant or negative association with good/high water status, this bias would only strengthen our conclusions. This database records locations of good fishing sites rather than information on the frequency of visits. We assume no *a priori* bias exists in frequency of use of these locations in relation to water status, once accounting for travel distance, population, income, access and substitutability (see “Statistical Analysis”).

### Outdoor Swimming Society’s wildswim.com Database:

This social-information website maintained by the Outdoor Swimming Society allows users to add swimming spots onto a geospatial map of the UK. Individual records are stratified to sea swimming, lake swimming, river swimming, lidos, tidal pools and estuaries. Many locations are supplemented by a brief description and additional user comments, however, recorded information is not officially verified in terms of reliability or accuracy of location. Similar to fishinginfo.co.uk one may assume that repeated visits by the same or multiple individuals would be recorded as a single record – which is also supported by the lack of similarly named and/or collocated records in the database. We therefore assume no *a priori* bias with respect to water status. Arguably, users of wildswim.com are unlikely to add spots if their swimming experience was unpleasant. Not surprisingly, the vast majority of descriptions of particular spots is positive (data not shown).
